# The value of serum neutralizing antibody in evaluating predictability of COVID-19 after recovery and the validation of vaccine

**DOI:** 10.1186/s12879-023-08465-9

**Published:** 2023-12-20

**Authors:** Bianqin Guo, Zheng Li, Gang Fu, Hong Li, Jing Yang, Zhenbin Zhang, Lixiang Wu, Jing Wang

**Affiliations:** 1 Department of Clinical Laboratory, Chongqing University Cancer Hospital & Chongqing Cancer Institute & Chongqing Cancer Hospital, Chongqing, China; 2 Tianjin Enterprise Key Laboratory of Chemiluminescence and POCT Diagnostic Technology, Tianjin, China; 3 Department of Clinical Laboratory, Chongqing Public Health Medical Treatment Center, Chongqing, China

**Keywords:** COVID-19, Neutralizing antibody, IgG, SARS‐CoV‐2

## Abstract

**Background:**

This work aimed to study natural humoral immune response to severe acute respiratory syndrome coronavirus 2 (SARS-CoV-2) infection.

**Methods:**

Chemiluminescent immunoassay (CLIA) was used to detect the neutralizing antibody (Nabs) and IgG.

**Results:**

Nabs peaked on days 57–96 after symptom onset and remained detected on days 97–132. The Nabs in the 32 patients who were dynamically monitored showed four changing patterns. The titers of Nabs and IgG were correlated, and three modes of relationship were found between them.

**Conclusions:**

Nabs showed a regular change in the course of coronavirus disease 2019 (COVID-19). The detection of Nabs is very important for monitoring the course of COVID-19 and predicting the strength of antibody protection.

**Supplementary Information:**

The online version contains supplementary material available at 10.1186/s12879-023-08465-9.

## Introduction

Severe acute respiratory syndrome coronavirus 2 (SARS-CoV-2), which causes coronavirus disease 2019 (COVID-19), is an envelope virus that includes Positive-sense single-stranded RNA (+ ssRNA), spike protein (S), membrane protein (M), envelope protein (E), and nucleocapsid protein (N) [1]. Spike protein is cleaved into S1 and S2 at furin and S2′ sites by specific proteases [2]. SARS-CoV-2 infects cells by the receptor binding domain (RBD) of S1 subunit binding to angiotensin-converting enzyme 2 (ACE2) receptor on the host cell membrane. Blocking the S protein of SARS-CoV-2 or the binding of its RBD to its receptor may help prevent the entry of the virus into cells, and thus, infection with SARS-CoV-2 is prevented [3]. Hence, the antibody that is aimed toward the S-RBD epitope of SARS-CoV-2 is named neutralizing antibody (Nabs); this could prevent SARS-CoV-2 from infecting cells by binding to the S-RBD epitope [4, 5].

Little is known about the nature and durability of the humoral immune response to infection with SARS-CoV-2. Many advantages have been found in Chemiluminescent immunoassay (CLIA) including strong specificity (https://www.bioscience-tj.com/productinfo/1341929.html; https://www.ncbi.nlm.nih.gov/pmc/articles/PMC8993648 (Sect. 2.2.1)), wide linear range, stable results, and simplified operation. By CLIA, we tested the Nabs levels that targeted S1-RBD to elucidate the persistence and model of change of Nabs in COVID-19 and rehabilitated patients. Our study showed that Nabs could maintain high levels in some rehabilitated patients at about 5 months after symptom onset. The change of Nabs was inconsistent in different COVID-19 patients. In some patients, the level of Nabs decreased significantly within a few months after recovery. Moreover, Nabs increased consistently after symptom onset or increased first and then decreased. In other patients, it first decreased and then increased. Nabs fluctuated during 60 days after the onset of symptoms.

## Materials and methods

### Patients

We identified 149 cases of COVID-19. The infection in the patients was confirmed by nucleic acid detection at the Chongqing Public Health Medical Treatment Centre between 29 January, 2020 and 8 June, 2020 for a retrospective study. Patients were divided into five groups according to the number of days after symptom onset. We monitored the levels of serum Nabs in 308 samples of the 149 cases. 122 cases of non-COVID‐19 served as the control group were collected between Feb 12, 2020, and Mar 30, 2020, from Chongqing University Cancer Hospital. We have access to information that could identify individual participants during and after data collection. The study protocol has been approved by the Ethics Committee of Chongqing Public Health Medical Rescue Centre and Chongqing University Cancer Hospital. All associated procedures were conducted in accordance with the approved guidelines. All participants provided written informed consent in accordance with the Declaration of Helsinki.

### SARS-CoV-2 nucleic acid assay

The patients’ nasopharyngeal swab samples and SARS-CoV-2 nucleic acid amplification were processed according to the instructions of kits from Da’an Biotechnology Co., Ltd, and Sansure Biotechnology Co., Ltd.

### Determination of Nabs and IgG for SARS-CoV-2

Serum samples were collected using anticoagulant-free vacuum blood collection tubes. Blood was centrifuged after complete coagulation, inactivated in a 56 °C water bath for 30 min, and stored at -20 °C until use. The IgG (cat.no.11,023) and Nabs (cat. no.11,027) against SARS-CoV-2 were detected in serum samples using SARS-CoV‐2 IgG Antibody kit and SARS‐CoV‐2 NAbs kit (Bioscience Diagnostic Technology, Tianjin) by a fully automatic chemiluminescence immunoassay analyzer from Bioscience (Axceed 260) according to the manufacturer’s instructions. To detect the IgG, first mix 50 µL of the IgG reagent one, 75 µL of the treated sample or negative control/positive control, 35 µL of the IgG reagent zero and 75 µL of the IgG reagent two. React for 35 min after mixing. Wash twice, add 200 µL of alkaline phosphatase chemiluminescence substrate solution to each tube after cleaning, protect from light, and read the reaction for 5 s. To detect the Nabs, 35µL of the Nabs reagent one, 50µL sample or negative control/positive control were added respectively, and 35µL of Nabs reagent reagent zero was added after 15 min of reaction. After cleaning, add 200 µL of alkaline phosphatase chemiluminescence substrate solution to each tube, protect from light, and read the reaction for 5 s. The target antigen used in the IgG kit was the S protein, and the target antigen used in the Nabs kit was the S1-RBD protein. The luminescence value of the samples was negatively correlated with Nabs. When the sample concentration was less than 2.0 AU/ml, the test result was negative. The specificity to IgG and Nabs were both 99%. lgG sample value/cutoff value (S/CO) = RLU of the sample/RLU of the cutoff value. S/CO < 1.0 resulted in a negative assay and vice versa. The cutoff value of the assay kit was defined as: cutoff = mean of positive control RLU × 0.1 + mean of negative control RLU. Parts of the antibody levels in the article were expressed as log2 (S/CO + 1). All tests were performed under strict biosafety conditions.

### Statistical analysis

GraphPad Prism software 8.0 was used for graphing and SPSS software 22.0 for statistical analysis. Median (interquartile) data was used for continuous variables, and Wilcoxon rank sum test was used for comparison between groups. A test of ɑ equal to 0.05 was used. The p ≤ 0.05 was considered as the significant difference.

## Results

### The performance of Nabs and IgG detected by CLIA

By CLIA, we analyzed the titers of IgG and Nabs in 308 serum samples of confirmed COVID-19 patients and 122 non-COVID-19 individuals. COVID-19 patients had a median age of 45 years, of whom 81 were female (Supplementary Table 1). Non-COVID‐19 patients had a median age of 53 years, of whom 72 were female (Table 1). The specificity for IgG and Nabs were 100.00% and 95.08%, respectively (Table 2).

**Table 1:** Demographic and clinical characteristics of enrolled patients

Characteristics	COVID-19	Non-COVID‐19
Number	149	122
Age, Median	45	53
Female	81	72
Male	68	50

**Table 2:** The specificity of IgG and Nabs reagent kits

Characteristics	IgG			Nab		
	COVID-19	Non-COVID‐19	COVID-19		Non-COVID‐19	
Samples	308	122	306		122	
Patients	149	122	147		122	
Positive results	133	0	139		6	
Specificity (%)	-	100.00%	-		95.08%	

### The antibody concentration trend of IgG and nabs in COVID-19 patients

**Fig. 1:**
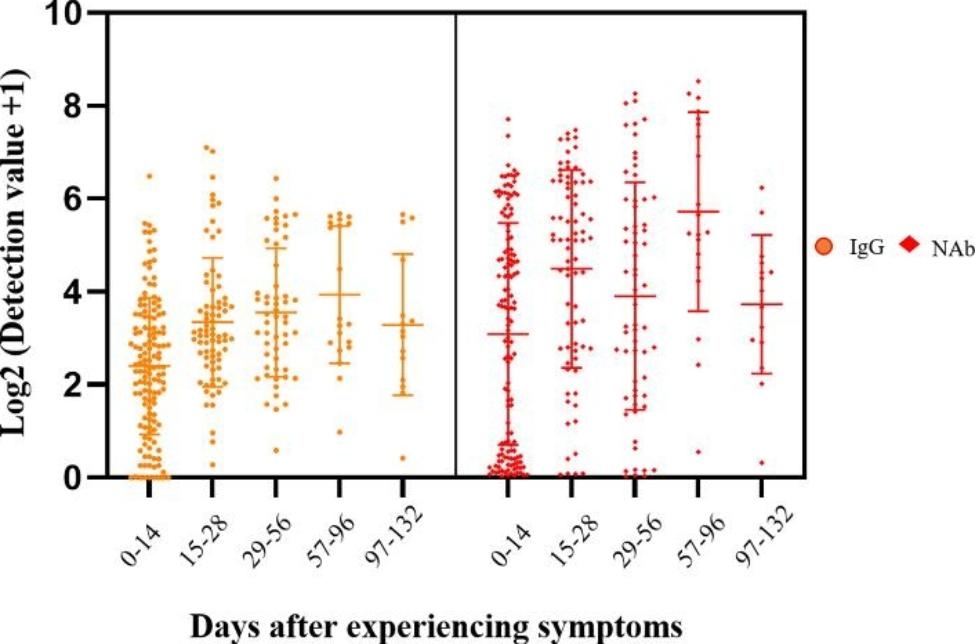
Titers of IgG and Nabs in different groups of COVID-19 patients

According to the time interval between sample collection and symptom onset, we divided all COVID-19 patients into five groups, namely 1-14d, 15-28d, 29-56d, 57-96d, and 97-132d (Fig. 1). The positive rate of IgG and Nabs detection showed an upward trend in the different groups. Among them, the percentage of IgG positive patients in the 29–56d group was lower than that in the 15–28d group. The main reason for this decline was as follows. In the cohort, five patients only had data from the 29-56d group and were IgG negative (Supplementary Table 2). The Nabs in the 29-56d group tested only 57 samples, of which 54 tested positive (Table 3), due to the limited volume of tested samples.

**Table 3:** The positive rate of IgG and Nabs in different groups of COVID-19 patients

Groups	*n*	IgG+					Nab+			
		*n*	%		Median	IQR	*n*	%	Median	IQR
0-14d	139	88	63.31%		9.47	33.83	97	69.78%	4.24	8.45
15-28d	75	69	92.00%		33.45	74.49	70	93.33%	7.40	7.58
29-56d	62	53	85.48%		13.83	57.28	54	94.74% (54/57)	10.11	26.72
57-96d	20	19	95.00%		43.55	171.09	19	95.00%	9.29	37.16
97-132d	15	14	93.33%		15.18	15.40	14	93.33%	7.87	13.43

### Changes and clinical significance of Nabs concentration in COVID-19 patients

**Fig. 2:**
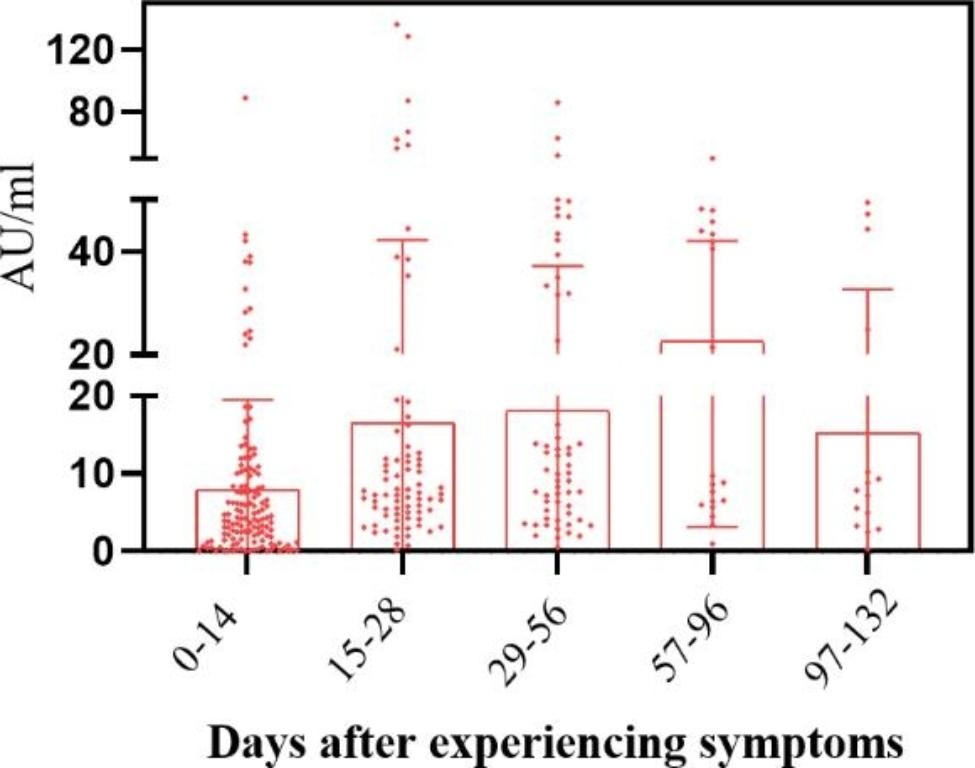
Titers of Nabs of confirmed COVID-19 patients in different groups

The titers of Nabs in COVID-19 patients who were confirmed to be positive by nucleic acid detection were measured by CLIA. Nabs level continued to increase during 96 days after symptom onset. Nabs titers reached the highest level in 57–96 days and then decreased. Even though the titers of Nabs began to decrease during 97–132 days after symptom onset, the level of Nabs was still high (Fig. 2).

**Fig. 3:**
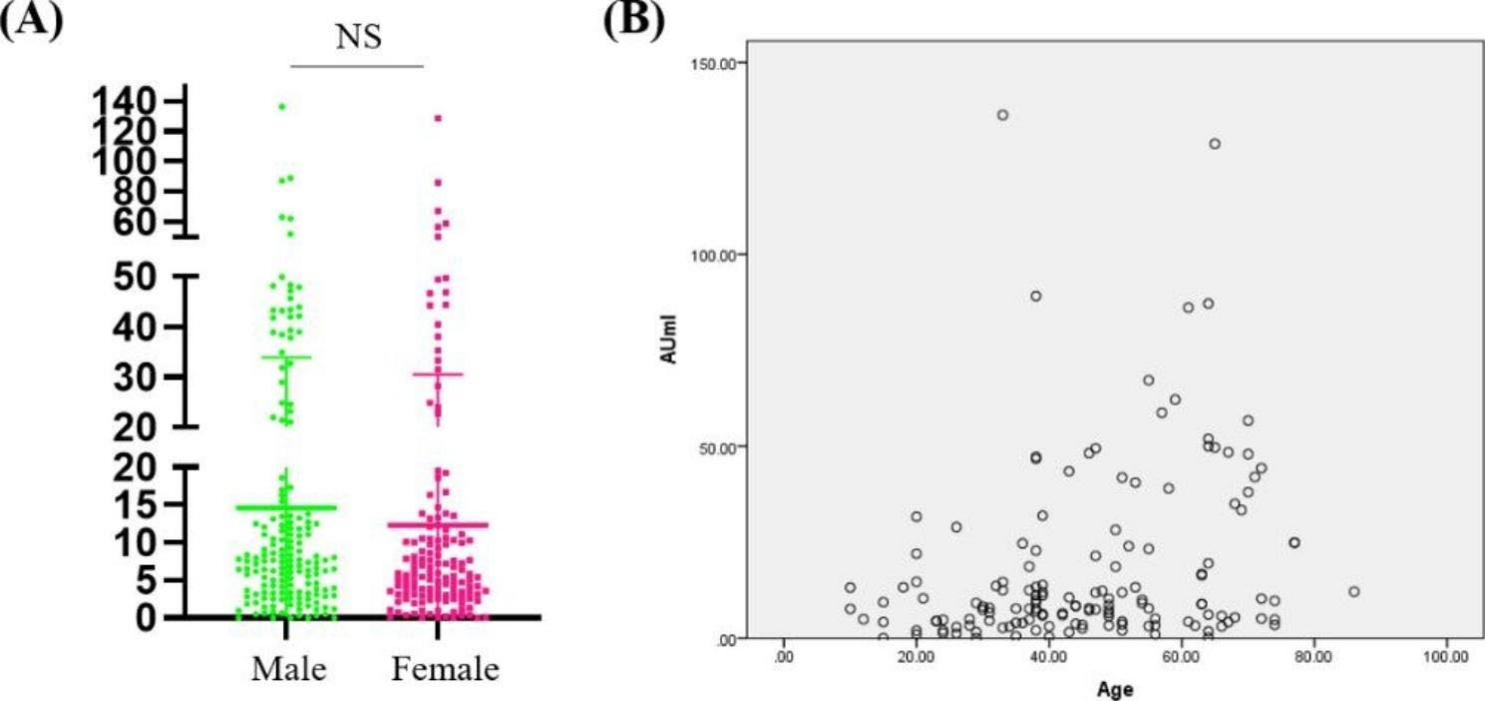
**A**. Titers of Nabs of confirmed COVID-19 patients in Male and female; **B**. The analysis of correlation with age and Nabs in COVID-19 patients

Male have higher levels of Nabs compared to female (Fig. 3A). Nabs levels and patient age were positively correlated (r = 0.263) (Fig. 3B).

The study by Khoury et al. showed that the Nabs level of 50% protection against detectable SARS-CoV-2 infection was 20.2% of the average recovery level. The level of Nabs required to provide 50% protection against severe infection was 3% of the average recovery period [11]. In this study, the start time of the disease recovery period was 26 days after the onset of symptoms. We counted the Nabs titer data for the 102 samples of the COVID-19 patients we collected from 26 days to 132 days. The aim was to explore the proportion of patients in our study subjects who achieved 50% protection during recovery (Table 4).

**Table 4:** Percentage of COVID-19 patients with 50% protection during the recovery period

Groups	n	> 20% of Mean	> 3% of Mean (Mean = 19.1AU/ml)
57-96d	20	90.0%	100.0%
97-132d	15	73.3%	93.3%

### Dynamic detection of changes in Nabs in COVID-19 patients

**Fig. 4:**
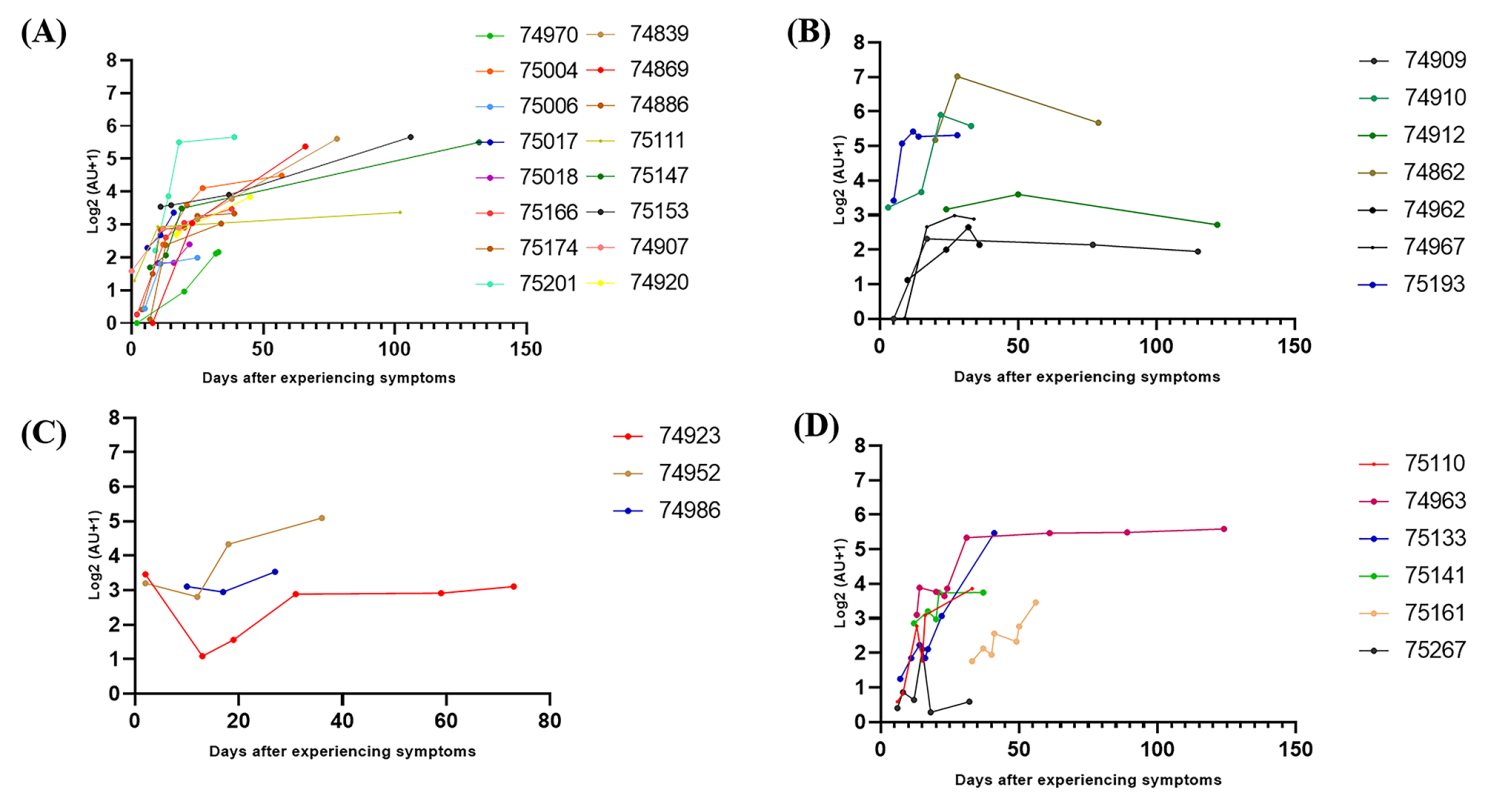
The dynamic change of Nabs of COVID-19 patients at different time points. (**A**) The level of Nabs was elevated during 0–132 days; (**B**) The level of Nabs first increased then decreased; (**C**) The level of Nabs first decreased then increased; (**D**) The level of Nabs repeatedly fluctuated

The clinical features and immune responses of individuals infected with SARS-CoV-2 have not been well described. Here, we dynamically detected Nabs in 32 COVID-19 patients and found four models. The first type of Nabs level continued to increase during the detection period. The second showed a trend of first increasing and then decreasing. In the third mode, the patient’s Nabs decreased and then continued to increase. Fourth, the Nabs remained at dynamic fluctuations (Fig. 4).

### The correlation between IgG and nabs in COVID-19 patients

**Fig. 5:**
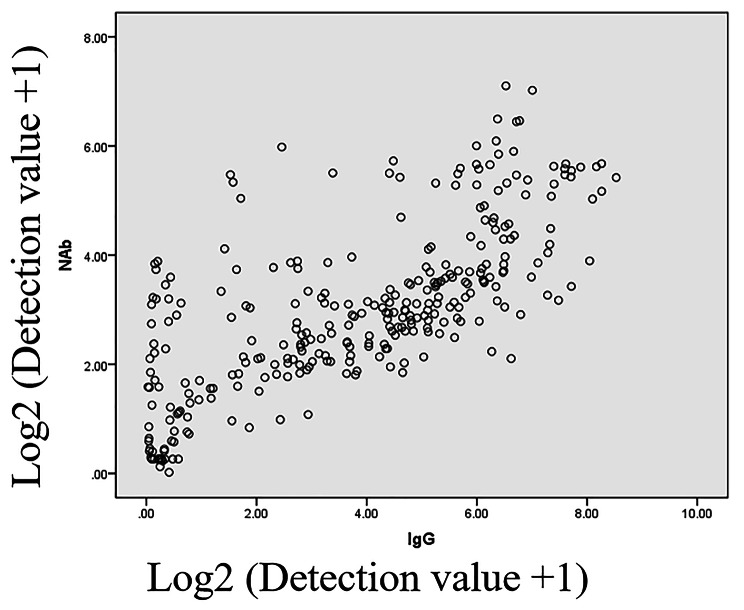
The analysis of correlation with IgG and Nabs in samples from SARS-CoV-2-infected patients

**Fig. 6:**
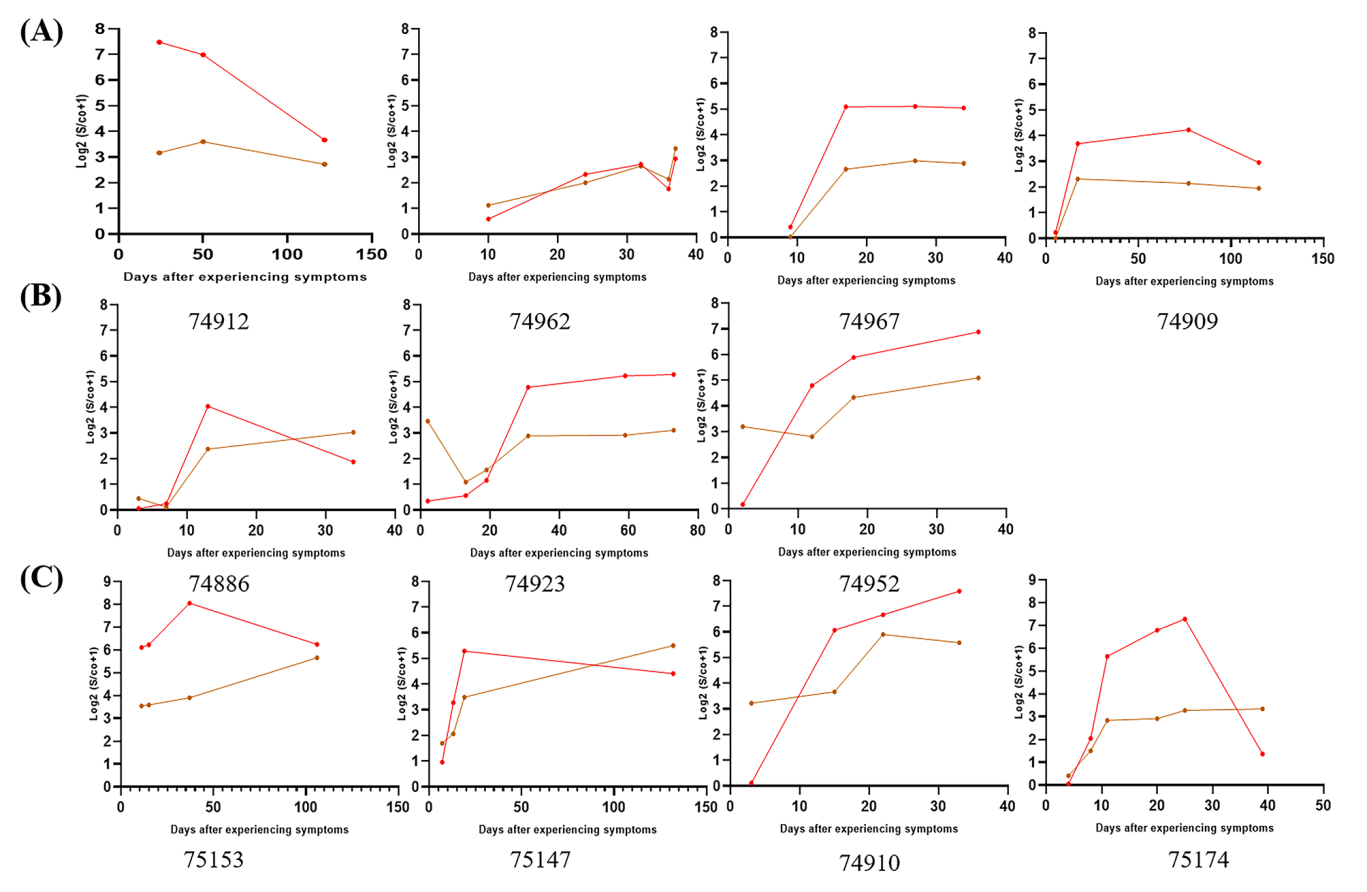
The dynamic change of Nabs and lgG in confirmed COVID-19 patients in different time points. **A**. The model of the dynamic change was completely consistent with IgG and Nabs. **B**. IgG was continuously increased; Nabs first decreased then increased. **C**. Nabs was continuously increased; IgG first increased then decreased

We performed a correlation analysis to investigate the correlation between Nabs and IgG. The results showed that Nabs and IgG were significantly correlated (r = 0.72, *p* < 0.01) (Fig. 5). We further studied the change rule of Nabs and IgG. Three change modes were identified. In the first one, Nabs and IgG changed synchronously. In the second, IgG continued to increase, and Nabs first decreased and then increased. In the third, IgG and Nab increased synchronously first; then, Nabs continued to increase, and IgG decreased (Fig. 6).

## Discussion

SARS-CoV-2 is a novel coronavirus that is transmitted primarily through direct contact and respiratory droplets or aerosols [6, 7]. Antibody-mediated humoral immunity is critical for the clearance of the virus and the prevention of recurring viral infections. After the body infected SARS-CoV-2, many antibodies are produced by humoral immune response, including non-Nabs and Nabs. Nabs may be IgG, IgM, and IgA, but not all IgG, IgM, and IgA are Nabs [8]. Non-Nabs could cause antibody-dependent enhancement and pro-inflammation. For example, IgG or IgM induced by nucleocapsid protein (N) could not prevent the virus binding to ACE2, which means this type of antibody could not play a neutralizing role. However, S1 epitope is the most popular for vaccination because of its neutralizing capacity. Some studies have shown the RBD subunit generated Nabs at the highest tier in rabbits among different S protein subunits, including S1, RBD subunit, S2, and modified variants [9]. In addition, RBD-modified SARS CoV-2 elicited a strong antibody response in rats [10]. Nabs that targeted the structural S1-RBD need to be studied further.

In this study, we evaluated the persistence of Nabs in convalescence patients of COVID-19. Nabs levels peaked between 57 and 96 days after symptoms appeared and remained high between 97 and 132 days. The time point in which Nabs had a significant reduction was not found in this study, we also havn’t get the result that how long Nabs could last on the body, but we showed that the Nabs in convalescence patients was still at higher levels for about 5 months and had a protective effect, thereby preventing reinfection. We analyzed the average level of Nabs in convalescent patients according to the method reported by Khoury DS et al. [11] to predict protection from the next infection and to assess the kinetics of weakened immunity over time. Compared with COVID-19 patients, the seroconversion level of Nabs in the vaccinated population was lower than that in COVID-19 patients, but the trend of concentration changes was similar to that in COVID-19 patients, peaking at 29–56 days (Supplementary Fig. 1). In addition, our study found no significant difference in Nabs titers with patient gender. This is not entirely consistent with the study by Markmann et al. [12]. The reason for this may be because we collected data from the early stages of infection to the recovery phase of the patients, and the published article was an analysis of patients in the recovery phase.

We dynamically tracked the change of Nabs at different time points in 32 COVID-19 patients during their illness and rehabilitation. There were four modes of change for Nabs. The first one was characterized by the continued increase in the level of Nabs within 0–132 days, which accounted for 50% (16/32). This finding was possibly due to the persistent release of a small amount of the virus in the body, causing a continuous increase in humoral immunity and resulting in a continuous increase in antibody levels. Another possibility was as follows: long-lived plasma cells may persist in the study subjects and continous secrete Nabs [13]. The second pattern was that the level of Nabs first increased and then decreased. In the initial stage of the disease, humoral immunity is enhanced due to the continuous stimulation of the virus, causing the level of Nabs to increase. With the clearance of the virus, the level of Nabs began to decrease accordingly. Another possibility is that the humoral immunity against SARS-CoV-2 came mainly from the extra-follicular immune response rather than from the classical Germinal Center Immune Response. SARS-CoV-2-specific Nabs is secreted by short-lived plasma cells, so the Nabs level gradually decreases. The third mode was that the level of Nabs was first reduced and then elevated, and the proportion of patients in this mode was the lowest (3/32). The initial Nabs level was higher in the third mode. The level of Nabs decreased quickly after neutralizing SARS-CoV-2. The virus was released again from the infected cells due to its incomplete clearance. The Nabs-specific plasma cells increased the secretion of Nabs, resulting in a continuous increase of Nabs. The fourth pattern was Nabs fluctuating repeatedly (6/32). This happened within 60 days of the onset of symptoms. Possibly, the virus was not cleared away completely. Accordingly, the reaction between the virus and the Nabs induced a fluctuation in the concentration of the Nabs. Another change modle of Nabs couldn’t be found, because of the numbers and time point for dynamically tracked samples not enough.

We analyzed the correlation between IgG and Nabs. IgG and Nabs had a significant correlation (r = 0.72). Furthermore, we analyzed the change patterns of IgG and Nabs. Three patterns were found. The change style of IgG was completely consistent with Nabs in nine patients, accounting for 56.25% (9/16); the second mode was the increase in IgG, but Nabs decreased first and then increased in three patients. From the figure, the inflection point of the Nabs’ decrease was at 10 days after the onset of symptoms. Nabs of type IgM and IgA could exist during this period. Nabs began to decrease when it neutralized the virus. The IgG-type Nabs included both neutralized IgG and non-neutralizing IgG. Thus, while the neutralizing IgG was consumed, the non-neutral IgG type gradually increased with the prolongation of the disease course. The third mode was the simultaneous increase of lgG and Nabs; then, they changed in different directions (4/16). The Nabs was elevated, and IgG decreased (1/4), or Nabs decreased, and IgG increased (3/4). It could be seen from the figure that the time point of the reverse happened at about 25 days after the onset of symptoms. IgG gradually increased during this time according to our results. We speculated that the main reason for the change in the opposite direction for IgG and Nabs was related to the change of ratio between linear phenotypic IgG and structural phenotypic IgG.

## Conclusions

In conclusion, Nabs are valuable for evaluating whether the virus has been cleared completely. It can be used for predicting the prognosis and the risk of reinfection. Nabs is very important for addressing SARS-CoV-2, because it prevents virus invasion. Our research is crucial for developing effective prevention and control strategies and reducing the recurrence of pandemics.

### Electronic supplementary material

Below is the link to the electronic supplementary material.

Supplementary Material 1

Supplementary Material 2

## Data Availability

The datasets used and analysed during the current study are available from the. corresponding author on reasonable request.

## Abbreviations

SARS-CoV-2: Severe acute respiratory syndrome coronavirus 2
CLIA: Chemiluminescent immunoassay
Nabs: Neutralizing antibody
COVID-19: Coronavirus disease 2019
+ssRNA: Positive-sense single-stranded RNA
RBD: Receptor binding domain
ACE2: Angiotensin-converting enzyme 2
