# Supplementary material for: The value of serum neutralizing antibody in evaluating predictability of COVID-19 after recovery and the validation of vaccine
Source: BMC Infect Dis. 2023 Dec 20;23:895. doi: 10.1186/s12879-023-08465-9 (PMC10734152; doi:10.1186/s12879-023-08465-9)

Supplementary table 2 The COVID-19 patients with only 29-56d test data

| ID | Groups（Day） | Titer |
| --- | --- | --- |
| 653475 | 29-56d (37) | 0.099 |
| 653453 | 29-56d (37) | 0.128 |
| 653284 | 29-56d (42) | 0.546 |
| 653142 | 29-56d (44) | 0.706 |
| 652999 | 29-56d (45) | 0.021 |

Supplementary figure 1 Titers of Nabs in different groups of vaccinated population


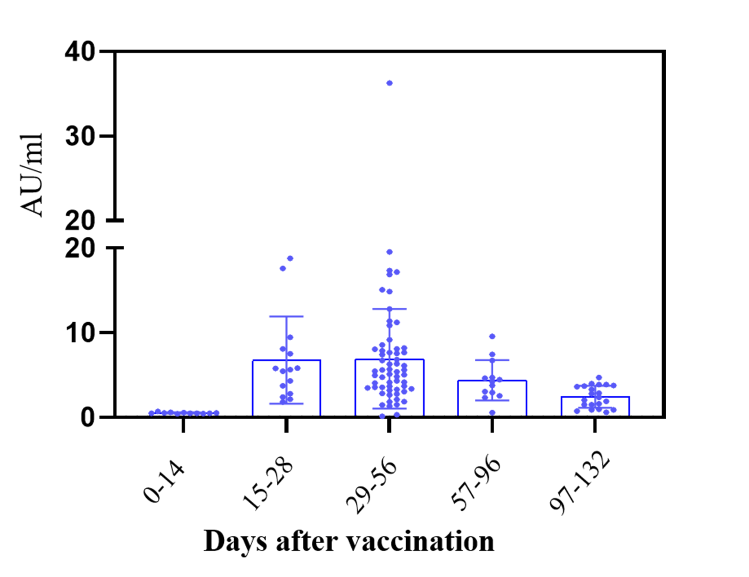

Supplement: Supplementary file 1 — Supplementary Material 1 [file 12879_2023_8465_MOESM1_ESM.docx]
